# 

**Published:** 1867-09

**Authors:** 


### CONTENTS.

ORIGINAL COMMUNICATIONS.

A Random Thought on Nitrous Oxyd.... 371
Principles and Practice of Filling Teeth.... 378
Duty of Dentists to Instruct their Patients respecting the care of the Teeth.... 381
Chicago Dental Society.... 383
Hereditary Influences on the Teeth.... 405

PROCEEDINGS OF SOCIETIES.

The Central Ohio Dental Association.... 408

EDITORIAL.

The Code of Ethics.... 410
Mad River Dental Society.... 412
Nitrous Oxyd Again.... 413
J. M. Brown.... 413
Reparatory.... 414
New Firm—Old Depot.... 414
Personal.... 414

